# Neuropathology in Mice Expressing Mouse Alpha-Synuclein

**DOI:** 10.1371/journal.pone.0024834

**Published:** 2011-09-26

**Authors:** Claus Rieker, Kumlesh K. Dev, Katja Lehnhoff, Samuel Barbieri, Iwona Ksiazek, Sabine Kauffmann, Simone Danner, Heinrich Schell, Cindy Boden, Markus A. Ruegg, Philipp J. Kahle, Herman van der Putten, Derya R. Shimshek

**Affiliations:** 1 Novartis Institutes for BioMedical Research, Novartis Pharma AG, Basel, Switzerland; 2 Department of Physiology, School of Medicine, Trinity College Dublin, Dublin, Ireland; 3 Neurobiology Biozentrum, University of Basel, Basel, Switzerland; 4 Department of Neurodegeneration, Hertie Institute for Clinical Brain Research and German Center for Neurodegenerative Diseases, University of Tübingen, Tübingen, Germany; Thomas Jefferson University, United States of America

## Abstract

α-Synuclein (αSN) in human is tightly linked both neuropathologically and genetically to Parkinson's disease (PD) and related disorders. Disease-causing properties *in vivo* of the wildtype mouse ortholog (mαSN), which carries a threonine at position 53 like the A53T human mutant version that is genetically linked to PD, were never reported. To this end we generated mouse lines that express mαSN in central neurons at levels reaching up to six-fold compared to endogenous mαSN. Unlike transgenic mice expressing human wildtype or mutant forms of αSN, these mαSN transgenic mice showed pronounced ubiquitin immunopathology in spinal cord and brainstem. Isoelectric separation of mαSN species revealed multiple isoforms including two Ser129-phosphorylated species in the most severely affected brain regions. Neuronal Ser129-phosphorylated αSN occured in granular and small fibrillar aggregates and pathological staining patterns in neurites occasionally revealed a striking ladder of small alternating segments staining either for Ser129-phosphorylated αSN or ubiquitin but not both. Axonal degeneration in long white matter tracts of the spinal cord, with breakdown of myelin sheaths and degeneration of neuromuscular junctions with loss of integrity of the presynaptic neurofilament network in mαSN transgenic mice, was similar to what we have reported for mice expressing human αSN wildtype or mutant forms. In hippocampal neurons, the mαSN protein accumulated and was phosphorylated but these neurons showed no ubiquitin immunopathology. In contrast to the early-onset motor abnormalities and muscle weakness observed in mice expressing human αSN, mαSN transgenic mice displayed only end-stage phenotypic alterations that manifested alongside with neuropathology. Altogether these findings show that increased levels of wildtype mαSN does not induce early-onset behavior changes, but drives end-stage pathophysiological changes in murine neurons that are strikingly similar to those evoked by expression of human wildtype or mutant forms.

## Introduction

Disorders collectively referred to as the α-synucleinopathies include a number of clinically diverse neurodegenerative diseases that constitute a critical biomedical problem. Prevalent α-synucleinopathies include idiopathic Parkinson's disease (iPD), dementia with Lewy bodies (DLB) (7–30% dementia in elderly), the Lewy body variant of Alzheimer's disease (LBVAD) with rare forms in some familial forms of PD (fPD), the familial form of AD and Down syndrome, multiple systems atrophy (MSA), Hallervorden-Spatz disease (HSD), neurodegeneration with brain iron accumulation type-1 (NBIA-1), Niemann-Pick Type C Disease (NPC), parkinsonism–dementia complex of Guam (PDC-Guam), diffuse neurofibrillary tangles with calcification (DNTC) and pure autonomic failure [1]. The common neuropathological hallmarks in neurons and glia are microscopic proteinaceous inclusions, composed mainly of aggregated fibrillar alpha-synuclein (αSN). αSN is an abundant presynaptic protein in the brain. Its 140 amino-acid sequence is highly homologous across human, rat and mouse (for review see [2]). Initially, αSN microscopic aggregates were postulated to play a key role in the pathophysiology of α-synucleinopathies. Neurotoxicity findings implicate αSN protofibrils, soluble αSN protein complexes, posttranslationally modified forms of αSN (in particular nitrosylated), phosphorylated at serine 129 (Ser129), as well as mono- and di-ubiquitinated αSN forms [3]. In DLB brains more than 90% of the insoluble αSN is phosphorylated at Ser129 compared to about 4% phosphorylated at Ser129 in brains of normal individuals. Furthermore, Ser129 phosphorylated αSN is targeted to mono- and di-ubiquitination in α-synucleinopathy brains [4]. Extensive phosphorylation at Ser129 and/or its mono- and di-ubiquitination are critical events in the pathophysiology of αSN. However, direct experimental evidence supporting this notion is lacking and it is still debated whether these molecular forms of αSN are on the critical pathophysiological path rather than representing molecular epiphenomena of the disease process.

As multiple toxic mechanism have been proposed for αSN, it is important to determine which of its molecular forms are on the critical pathophysiological path. One main hypothesis of αSN toxicity is based on its capability to form toxic oligomers. Familial forms of Parkinson's disease possess mutant forms of αSN A53T and A30P (and E46K) that form oligomers more rapidly than wildtype αSN. In idiopathic forms of α-synucleinopathies that lack heritable αSN mutations, it is speculated that compromised handling of αSN and/or specifically modified forms are hampering αSN catabolism as well as that of other proteins. Oxidative damage of αSN could change αSN into toxic forms that trigger such a pathophysiological cascade [3].

It is unclear how critical to the disease process are some of the differences in αSN amino-acid sequence between human, rat and mouse. There is no solid evidence for endogenous mouse αSN co-aggregating with human αSN expressed in transgenic rodent models [5], [6], [7], [8], [9]. Furthermore, non-fibrillar αSN neuropathology in brain regions of human αSN transgenic mice is prominent also in regions where neurons express little or no endogenous mouse αSN [6], [7]. Some transgenic mouse models develop human-like fibrillar αSN structures and this may to a large extent depend on the transgene expression cassette that is used [7]. Thus, it appears that αSN pathology in transgenic species varies and is influenced by a number of experimental and endogenous factors. Knowing these factors could shed more light on genetic and environmental risk factors associated with diseases involving αSN. In an attempt to resolve some of these questions we generated transgenic mice over-expressing murine wildtype αSN driven by the Thy1 regulatory sequences enabling a direct comparison with previous human αSN transgenic lines generated previously in our laboratory [6].

## Results

### High expression of mαSN mRNA and protein levels in the Thy1-mαSN transgenic mouse line

**Figure 1:**
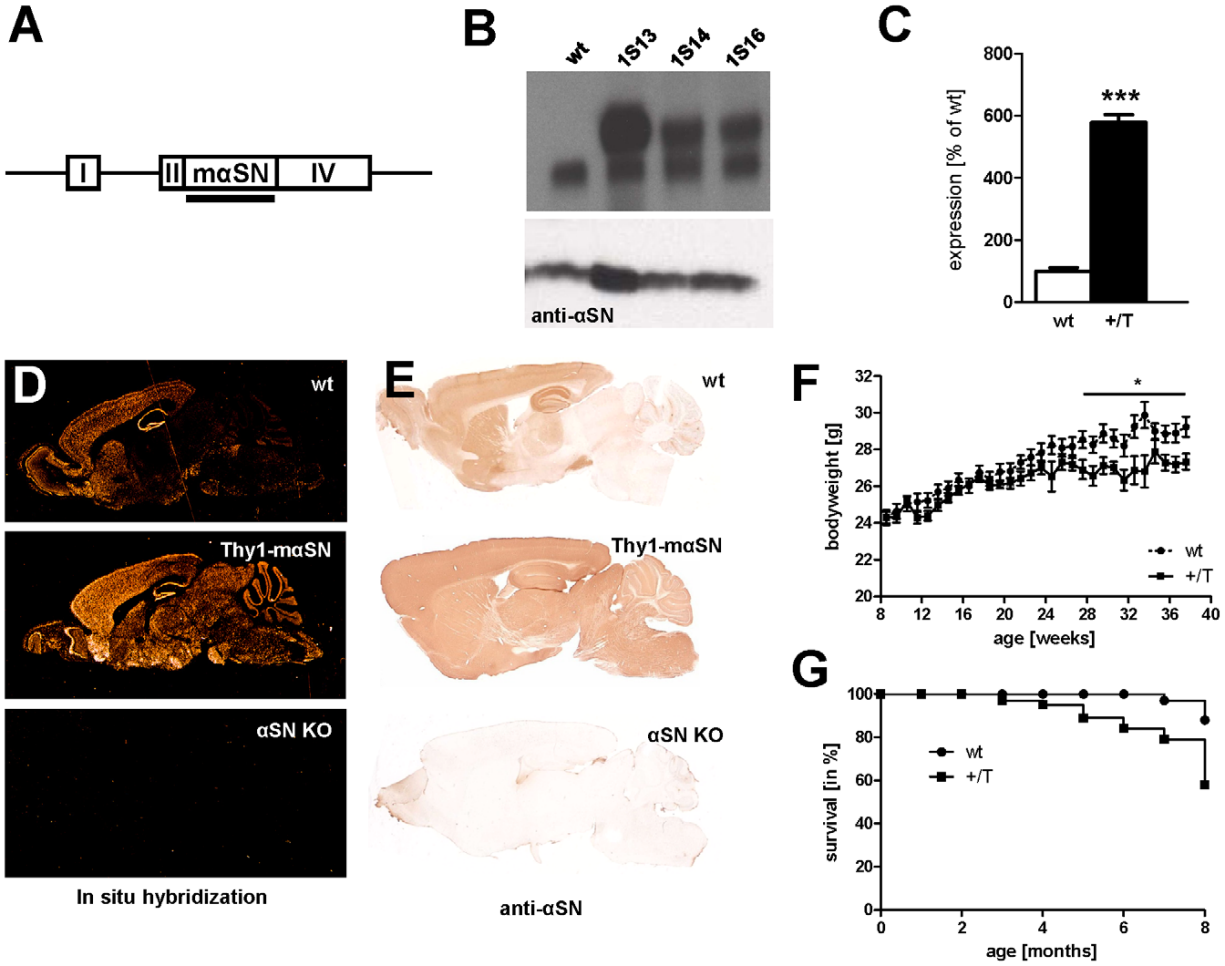
Thy1-mαSN and wildtype mice transgene expression. (**A**) Schematic diagram depicting wildtype murine αSN expression under the control of Thy1 regulatory sequences. *Roman numerals* refer to murine Thy1 exons. A filled horizontal *bar* indicates the mouse αSN cDNA probe that was used for northern and the cRNA probe used for *in situ* hybridization. (**B**) **(upper part)** Northern blot analysis of 10 µg total brain RNA per lane showing wildtype (wt) and mice from the Thy1-mαSN mouse founder lines 1S13, 1S14 and 1S16. **(lower part)** Immunoblot analysis using a monoclonal anti-αSN antibody showing the highest over-expression of murine αSN protein in the founder line 1S13, as compared to wt, founder mouse lines 1S14 and 1S16. (**C**) Quantification of over-expressed αSN protein in offsprings of the founder line 1S13 (+/T, black) compared to wt (white). wt: n = 7; +/T: n = 13. Data are shown as mean ± SD. (**D**) *In situ* hybridization in sagittal brain sections of a wt mouse, a Thy1-mαSN (line 1S13) and a αSN knock-out (KO) mouse. Probe was a ^35^S-labeled mouse αSN cRNA as represented by bar in Fig. 1A. (**F**) Free-floating sagittal whole brain sections immunostained for murine αSN in wt, Thy1-mαSN (line 1S13) and αSN KO mouse. (**G**) Body weight assessment in wt littermates (dotted, n = 12) and Thy1-mαSN (+/T, black, n = 10, line 1S13). (**H**) Thy1-mαSN (+/T) transgene mice show increased mortality rate compared with wt littermates (n = 10). * p<0.05; *** p<0.001.

Three transgenic C57BL/6 mouse lines were produced that express different levels of the Thy1-mouse αSN (mαSN) transgene (**Figure 1A**). Two lines *1S14* and *1S16* had comparable low levels whilst the third line *1S13* expressed transgene mRNA (**Figure 1B** **upper part**) and protein (**Figure 1B** **lower part**) levels in brain that were up to 6-fold above endogenous αSN in wildtype mice as shown by quantification in **Figure 1C**. Transgene mRNA levels in *1S13* mouse line were comparable to those in two lines described previously, expressing the A53T fPD and wildtype form of human αSN (hαSN) [6]. Our analyses described here focus on the line *1S13* (named Thy1-mαSN hereafter).

Similar to the Thy1-hαSN mouse lines [6], expression of Thy1-mαSN transgene mRNA and mαSN protein in Thy1-mαSN mouse brain was widespread. This is illustrated by in situ hybridization (**Figure 1D**) and αSN protein immunohistochemistry (**Figure 1E**) in low-magnification sagittal brain sections from Thy1-mαSN (αSN knock-out (KO) mouse brains served as a negative control). The overall expression pattern of the transgene in Thy1-mαSN was also very similar to those reported for the two lines expressing hαSN under the control Thy1 regulatory sequences [6]. Interestingly there was no apparent weight loss in Thy1-mαSN mice until 6 months of age (**Figure 1G**) in contrast to mice over-expressing hαSN with an early-onset weight loss (**Figure S1A**). Not until around 6–7 months of age Thy1-mαSN mice stopped gaining weight and in addition start to display severe motor deficits. This is again in sharp contrast to Thy1-hαSN mice that showed early-onset impairments of motor performance (**Figure S1 and** [6]). Furthermore we observed increased mortality in Thy1-mαSN mice compared to control wildtype (wt) littermates (**Figure 1H**).

### Overexpression of wildtype murine αSN leads to mild impairment of motor performance

**Figure 2:**
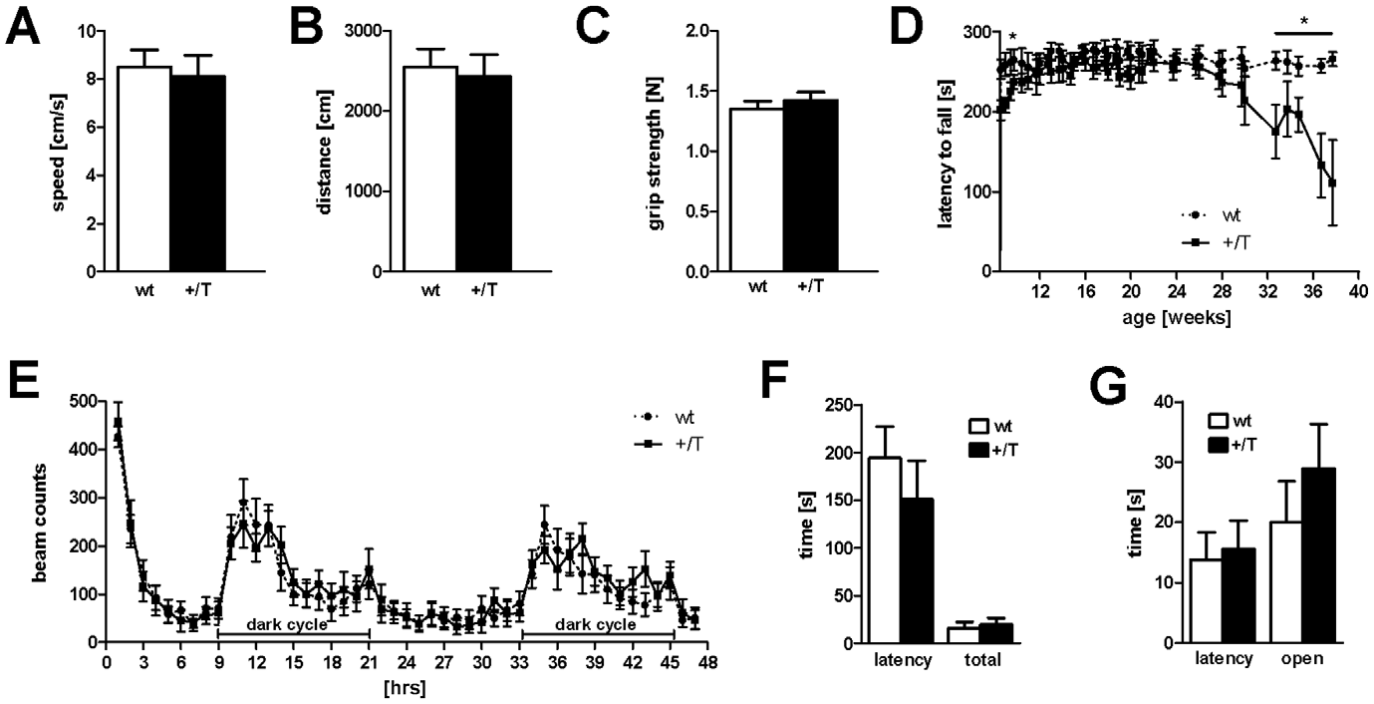
Behaviour analysis of Thy1-mαSN mice. (**A**) Measurement of the velocity and the total distance (**B**) in the open field paradigm revealed no change between Thy1-mαSN (+/T, black, n = 10) and wildtype (wt, white, n = 10) mice. (**C**) No difference in the forelimb grip strength between Thy1-mαSN and wt mice. (**D**) At the age of 28 weeks we observed a decline in performance of Thy1-mαSN (+/T, black line) compared to wt (dotted line) mice assessed by the accelerating rotarod. (**E**) Thy1-mαSN mice show similar activity pattern as wt mice over a period of 48 h (dark periods are indicated). (**F**) Quantification of the latency of the first entry to the dark compartment and of the time spent in the lit compartment in the dark/light box in 5 min. Thy1-mαSN and wt. (**G**) Measurement of the latency of the first entry to the open arms and the time spent in the open arms of the elevated plus-maze in 5 min. Thy1-mαSN and wt. Age of the animals: 6 months. Data are shown as mean ± SEM (n = 12). * p<0.05.

We performed different behavioral studies to determine motor function. Thy1-mαSN mice showed no difference in the open field paradigm. Neither velocity (**Figure 2A**) nor total activity (**Figure 2B**) was changed. Furthermore, no difference could be detected in forelimb grip strength (**Figure 2C**). Motor coordination was assessed using the accelerated rotarod task starting at two months of age. During the first four weeks, Thy1-mαSN mice showed impaired motor learning but by 12 weeks of age and after a number of training sessions, the performance of Thy1-mαSN mice was indistinguishable from wt mice up to the age of six months (**Figure 2D**). From 6–7 months onwards, a steady and rapid decline in rotarod performance in Thy1-mαSN mice became obvious (**Figure 2D**). Interestingly no difference in light/dark cycle activity, assessed by an actimeter for 48 h, could be detected between Thy1-mαSN and wt mice (**Figure 2E**). In order to determine the anxiety of Thy1-mαSN mice we performed dark-light box and elevated plus maze experiments (**Figure 2F,G**). We observed similar latencies and total time spend in the lit compartment between wt and mutants in the dark-light box (**Figure 2F**), suggesting no impact on anxiety. This was fortified using the elevated plus maze (**Figure 2G**). It is remarkable that Thy1-mαSN mice displayed a late-onset and much less pronounced motor impairment than transgenic mice expressing the hαSN transgene with early-onset (already at 5 weeks of age) and steady decline in motor control (**Figure S1 and** [6]).

### Perikaryal and neuritic accumulation of mαSN

Similar to earlier observations in mice expressing hαSN forms [6] we found mαSN expressed in many neurons in telencephalon, hippocampus, brainstem, cerebellar nuclei and spinal cord (**Figure 1E**). The mαSN expression in the hippocampus showed an increase in perikaryal and neuritic immunostaining for αSN and cerebellar nuclei respectively (**Figure S2**). In a substantial neuronal subset expression of the transgene was sufficient for perikaryal and neuritic mαSN accumulation, which did not change over time (**Figure S2**). This is further demonstrated by mαSN immunostaining of hippocampal neurons in mice expressing the Thy1-mαSN transgene on a mouse genetic background with a disrupted endogenous αSN gene (αSN KO) (**Figure S3D**). The specificity of the αSN immunostainings is illustrated by the very low levels of background staining in αSN KO mouse brain sections (**Figure S3C**).

### Prominent development of mαSN pathology, axonal degeneration and breakdown of myelin sheaths in spinal cord and brainstem

**Figure 3:**
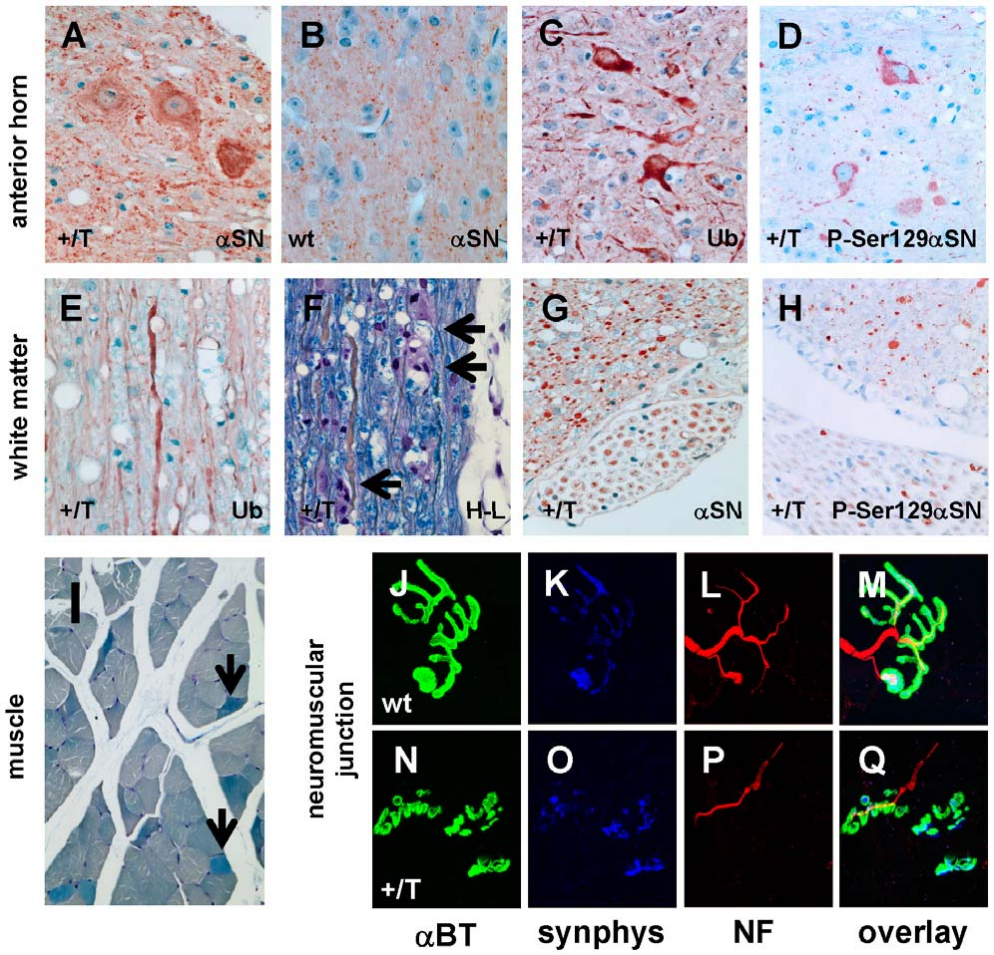
Pathology in Thy1-mαSN mouse spinal cord. (**A–D**) Correspond to sections through the anterior horn. Prominent perikaryal and neuritic mαSN staining (5038, Chemicon) in Thy1-mαSN (+/T) (**A**) but not in wildtype (wt) (**B**) mouse spinal cord section. Expression of endogenous αSN, same antibody as in (**A**), is restricted to synaptic boutons in wt mice (**B**). Strong ubiquitin (Ub) immunolabeling in spinal motor neurons, some with spindle-shaped dilatations of proximal dendrites (**C**). Some anti-P-Ser129αSN immunoreactive motor neurons and presynaptic boutons in transgenic (**D**) but not wt mice (not shown). (**E–H**) representative images of the spinal cord white matter including spinal roots in longitudinal sections (**E,F**) and cross sections (**G,H**). Many axons stained with anti-ubiquitin (Ub) (**E**). Holmes-Luxol stain depicting axonal degeneration in long white matter tracts of the spinal cord and breakdown of myelin sheaths with ovoids (arrows) (**F**). Many spinal cord and root axons immunolabel using anti-αSN antibody (5038, Chemicon) (**G**). A subset of central and peripheral axons was immunolabeled with anti-P-Ser129αSN antibody (**H**) that showed no staining in wt mice (not shown). Central axons are often enlarged (**G,H**). (**I**) Cross-sectioned muscle fiber bundle with small angulated fibers (arrows). (***J–Q***) Soleus neuromuscular junctions from wt (**J–M**) and Thy1-mαSN (**N–Q**) mice. Neuromuscular junctions were stained with α-bungarotoxin (αBT) to label postsynaptic acetycholine receptors (**J,N**), synaptophysin (synphys) (**K,O**), and neurofilament (NF) (**L,P**). Overlay of all three colors is shown (**M,Q**).

Like Thy1-hαSN mice [6], the Thy1-mαSN mouse developed a pronounced αSN pathology in spinal cord around the age of 6 months. We located prominent perikaryal and neuritic αSN staining in sections through the anterior horn (**Figure 3A,B**) and in addition strong ubiquitin immunoreactive motor neurons with spindle-shaped dilated proximal dendrites (**Figure 3C**). Using an antibody specific for the serine 129 phosphorylated form of αSN (P-Ser129αSN), we found immunolabeling of motor neuron cell bodies and presynaptic boutons in transgenic (**Figure 3D**) but not wt mouse spinal cord (not shown). The P-Ser129αSN antibody recognizes specifically a form of αSN that is phosphorylated at Ser129 and is abundant in α-synucleinopathy lesions in the diseased human and αSN transgenic brain, but not in normal mouse brains [10].

Until recently αSN axonal pathology was grossly underestimated although it is now documented in several of the transgenic animal models [6], [7], [11], [12], [13]. In this current study, we found that motor neuron pathology was accompanied by axonal pathology in spinal cord white matter (**Figure 3E–H**). Immunostaining for ubiquitin (**Figure 3E**), and Holmes-Luxol staining (**Figure 3F**) revealed axonal degeneration in long white matter tracts of the spinal cord with breakdown of myelin sheaths into rows of myelin ovoids. Many axons in the cord and spinal roots were immunolabeled with αSN antibody (**Figure 3G**). Central axons were often enlarged and a subpopulation immunolabeled with anti-P-ser129αSN (**Figure 3H**) which consistently left unstained the same tissue in wt mice (not shown). Unlike in the transgenic lines expressing hαSN [6], ubiquitin immunopathology was detected in every single Thy1-mαSN mouse aged 6 months (see below).

The αSN histopathology in brainstem, cerebellum and spinal cord was accompanied by prominent astrogliosis (**Figure S4A–E**), microgliosis (IBA1-positive cells; **Figure S4F–H**) and axonal degeneration (Campbell silver stainings; **Figure S4I–K**). Notably, other brain areas including hippocampus, cortex, striatum and thalamus showed little or none of these histopathological hallmarks despite many neurons showing high αSN transgene expression in these brain areas.

### Synaptic defects in the neuromuscular junction

Aggregates and/or soluble forms of αSN are present in neuronal somata and dendrites under pathological conditions in human and αSN transgenic mouse brains as well as in cultured neurons [14]. This contrasts with αSN being mainly presynaptic under normal circumstances. With respect to adverse effects on the neuronal cell in its entirety, it remains unclear whether pre- or post-synaptic changes are compromised first. Interestingly, muscles contained small angulated fibers reminiscent of neurogenic muscular atrophy (**Figure 3I**). In addition, we found that neuromuscular synapses showed signs of presynaptic degeneration although less pronounced as reported previously in lines expressing hαSN [6]. α-Bungarotoxin staining patterns for postsynaptic acetylcholine receptors were not different between wt and Thy1-mαSN mice (age 6 months) (**Figure 3J,N**), neither in soleus (slow-twitch, **Figure 3J–Q**) nor extensor digitorum longus (EDL, fast-twitch) muscles (not shown). Also, we detected little or no changes between wt and transgenic mice in presynaptic synaptophysin staining (**Figure 3K,O**). In contrast, staining of presynaptic neurofilaments differed dramatically. The neuromuscular junctions in Thy1-mαSN mice showed thinning or absence (not shown) of presynaptic neurofilament staining (**Figure 3L,P**). In summary, neuromuscular junctions in Thy1-mαSN mice showed degeneration that was independent of muscle fiber type and similar, as reported for mice expressing hαSN transgene [6].

### Phosphorylated αSN is expressed in hippocampal neurons that lack ubiquitin pathology

**Figure 4:**
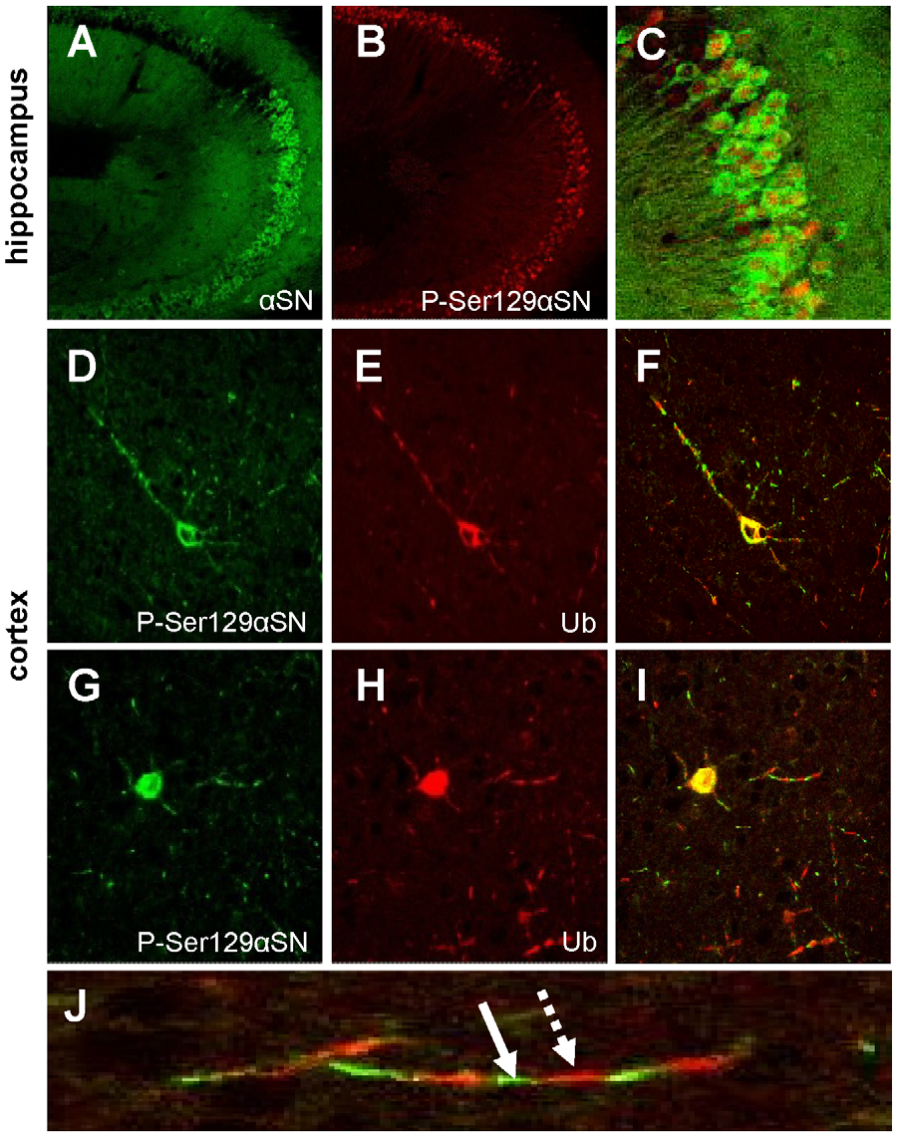
Co-localisation of Ubiquitinated and Phosphorylated mαSN. (**A–C**) confocal images of immunostains for αSN (S63320, Transduction Labs) (**A**), P-Ser129αSN (**B**) and the high power overlay (**C**) in adult Thy1-mαSN mouse hippocampus. (**D–I**) Confocal images of two P-Ser129αSN (**D,G**) and Ubiquitin (Ub)-immunopositive neocortical neurons (**E,H**) showing overlapping distribution patterns in cell soma (**F,I**), but a largely non-overlapping distribution pattern of the epitopes in processes as highlighted by the enlargement (**J**). The broken arrow indicates an Ub-immunostained zone (red, Cy3), the solid arrow a flanking region on the same process that is strongly immunopositive for P-Ser129αSN (green, FITC).

Strong αSN immunoreactivity could be observed in perikarya and dendrites, mainly in the CA3 region of the hippocampus (**Figure 4A**). Immunostaining with anti-P-Ser129αSN showed the abundant presence of the phosphorylated form of αSN in many neurons and throughout brain regions where the transgene is expressed (**Figure 4B,C**). In the hippocampus, a significant subset of neurons showed perikaryal and dendritic accumulation of αSN (**Figure 4C**). The more striking observation was that in CA1 and CA3 hippocampal neurons, when co-immunolabeled for αSN and P-Ser129αSN, the latter was prominently localized in the nucleus (**Figure 4C**). Similar to a previous study [6], the hippocampus lacked neurons immuno-positive for ubiquitin (not shown). Also in cortex only very few neurons showed ubiquitin pathology and/or a perikaryal accumulation of αSN (**Figure 4E,H**).

### Non-overlapping phosphorylated αSN and ubiquitin puncta along neuronal processes

Double labeling for ubiquitin and P-Ser129αSN was carried out in paraffin sections of neurons in regions such as the cortex, where only very few cells stained for ubiquitin (**Figure 4D–I**) and additionally in regions with pronounced ubiquitin pathology such as brainstem, colliculus and spinal cord (not shown). This revealed an extraordinary staining pattern, in particular, in processes. As shown, P-Ser129αSN and ubiquitin immunopositive stretches in processes alternate and did not overlap (**Figure 4J**). In contrast, in cell somata, the distribution patterns of P-Ser129αSN and ubiquitin were strikingly similar and overlaped to a high extent (**Figure 4D–I**).

### Thy1-mαSN mice displayed abnormal mitochondria, demyelination, axonal loss and non-fibrillar amorphous aggregates

**Figure 5:**
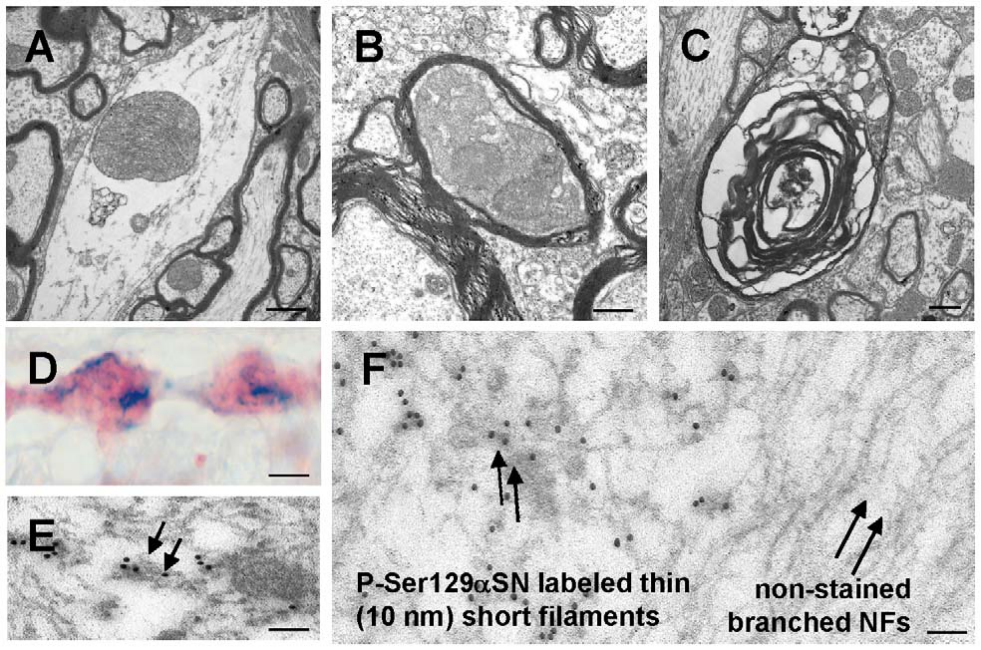
Ultrastuctural features of neurons in Thy1-mαSN mice. (**A**) Section through the anterior horn of the spinal cord. (**B**) Spinal cord white matter containing a cross-sectioned myelinated nerve fiber. (**C**) End-stage axonal degeneration. (**D**) detail of a longitudinal 4 µm thick paraffin section through the spinal cord after dual labelling with affinity purified anti-αSN (red, 5038, Chemicon), and anti-P-Ser129αSN (blue). (**E,F**) Immunoelectron microscopic images of white matter axons in the spinal cord in 6 months old Thy1-mαSN animal. The dark particles are 10 nm colloidal gold conjugated to the P-Ser129αSN-specific antibody. (**E**) In the right side of the photograph are negatively stained neurofilaments, arrow-head shows side-branches protruding from the filaments. Seen on the left are filaments focally decorated by gold particles and devoid of side-branches, arrows show non-fibrillar amorphous aggregates, as shown in greater detail in **F**. Scale bars: A, 1.7 µm (4,500× magnification); B, 0.4 µm (20,000× magnification); C, 0.6 µm (12,000× magnification); D, 20 µm (100× magnification); E–F, 0.25 µm (30,000× magnification).

Enlarged mitochondria are a sign of cells trying to compensate for energy deficits, reflecting local increases in the need for energy, vacuolization and/or loss of inner-outer membrane integrity of the mitochondria. We found grossly enlarged mitochondria with an abnormal high number of cristae but without any obvious vacuolization in spinal cord dendrites of Thy1-mαSN mice (**Figure 5A**). Spinal cord axonal degeneration was also evident by an accumulation of pathological organelles (including mitochondria) in the axoplasma (**Figure 5B**) with disappearance of the axon, loosening of the myelin wraps and vesicular disruption of the myelin sheath (**Figure 5C**). Interestingly, the axon showed pronounced beading with focal anti-P-Ser129αSN staining on a background of diffuse αSN immunostaining (**Figure 5D**). Immunoelectron microscopy (10 nm immunogold) showed P-Ser129αSN antibody-negative immunostained neurofilaments with side-branches protruding from the filaments in Thy1-mαSN (**Figure 5E**). We found that αSN over-expression results in short, thick, and less well oriented filaments of approximately 10 nm in diameter. They were devoid of side-branches, focally decorated by gold particles and coincide with non-fibrillar amorphous aggregates (**Figure 5E,F**) similar as the granular aggregates found in Thy1-hαSN mice [6].

**Figure 6:**
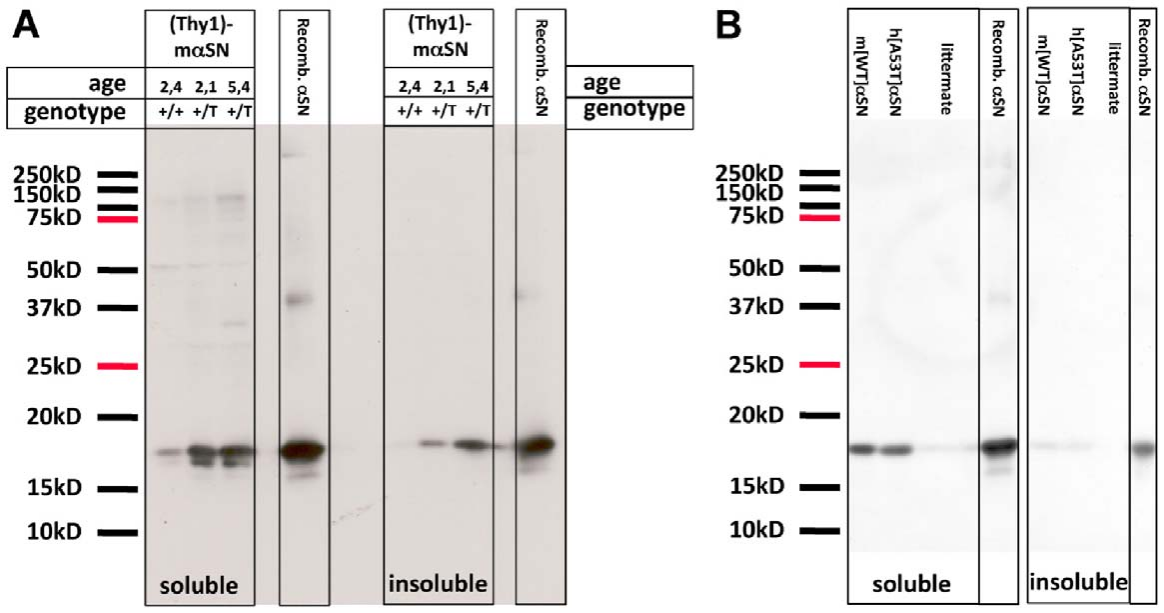
Solubility assays of Thy1-mαSN mouse brain tissue. Pooled frozen brainstems from 3-5 mice (equal 0.2 g wet weight) were homogenized in Tris buffer. Cleared supernatant were centrifuged for 20 min at 350.000× *g*. The buffer-soluble supernatants were loaded directly (“soluble”). Pellets were dissolved in Tris buffer containing 1% Triton X-100. After another centrifugation for 20 min at 350.000× g, the supernatants were loaded (“insoluble”). Western blots were prepared and probed with anti- αSN. (**A**) Samples from average 2.4 months old wildtype littermate mice (+/+) showed the endogenous αSN band in the buffer-soluble fractions. In addition, heterozygous Thy1-mαSN mice (+/T) showed αSN monomeric bands also in the buffer-insoluble fractions. Older (average 5.4 months old) mice showed more αSN in the Triton X-100 fraction than young (average 2.1 months old) mice. (**B**) The amounts of soluble and insoluble αSN in 5–6 months old heterozygous Thy1-mαSN mouse brain samples were comparable to those in age-matched heterozygous Thy1-h[A53T]αSN mice. Positions of molecular weight markers are indicated to the left, purified recombinant αSN (10 ng) yielded control signals and co-migrated with the brain-derived bands. Results are representative of 3 independent extractions involving a total number of 42 mice (**A**) and 2 independent extractions with 17 mice total (**B**), respectively.

To provide biochemical evidence for the observed aggregation of αSN in Thy1-mαSN mice, we perfomed solubility assays (see [13]). Brainstems from 3–5 mice were pooled, yielding matched starting tissue wet weight of 0.2 g. Tissue was homogenized in Tris buffer, and the buffer-insoluble material was dissolved in 1% Triton X-100. Such fractions were immunoblotted and probed with anti-αSN. The endogenous αSN from wt mice was mostly recovered in the buffer-soluble fraction, as expected (**Figure 6**). Transgenic mice showed the increased expression of αSN in the buffer-soluble fraction. In addition, Thy1-mαSN mouse tissues contained also buffer-insoluble αSN (**Figure 6**). Importantly, and consistent with the age-dependent aggravation of neuropathology (see above), the amount of buffer-insoluble αSN increased when comparing 2–3 months with 5–6 months old mice (**Figure 6A**). Thus, the increase of insoluble αSN in these mouse brains was not simply due to higher total αSN expression, but seems to indicate a shift towards insolubility with age. The amounts of soluble and insoluble αSN in 5–6 months old heterozygous Thy1-mαSN mouse brain samples were comparable to those in age-matched heterozygous Thy1-h[A53T]αSN mice (**Figure 6B**).

No SDS-PAGE resistant higher molecular weight smears were detected in the insoluble fractions. Further analysis of the detergent-insoluble material showed no detectable αSN in sarcosyl extracts (not shown), where fibrillar “amyloid” αSN would be expected. Taken together, the histological and biochemical analyses revealed insoluble, non-fibrillar aggregates in Thy1-mαSN mouse brains.

### Various post-translational modified αSN isoforms expressed in neurons of Thy1-mαSN mice

**Figure 7:**
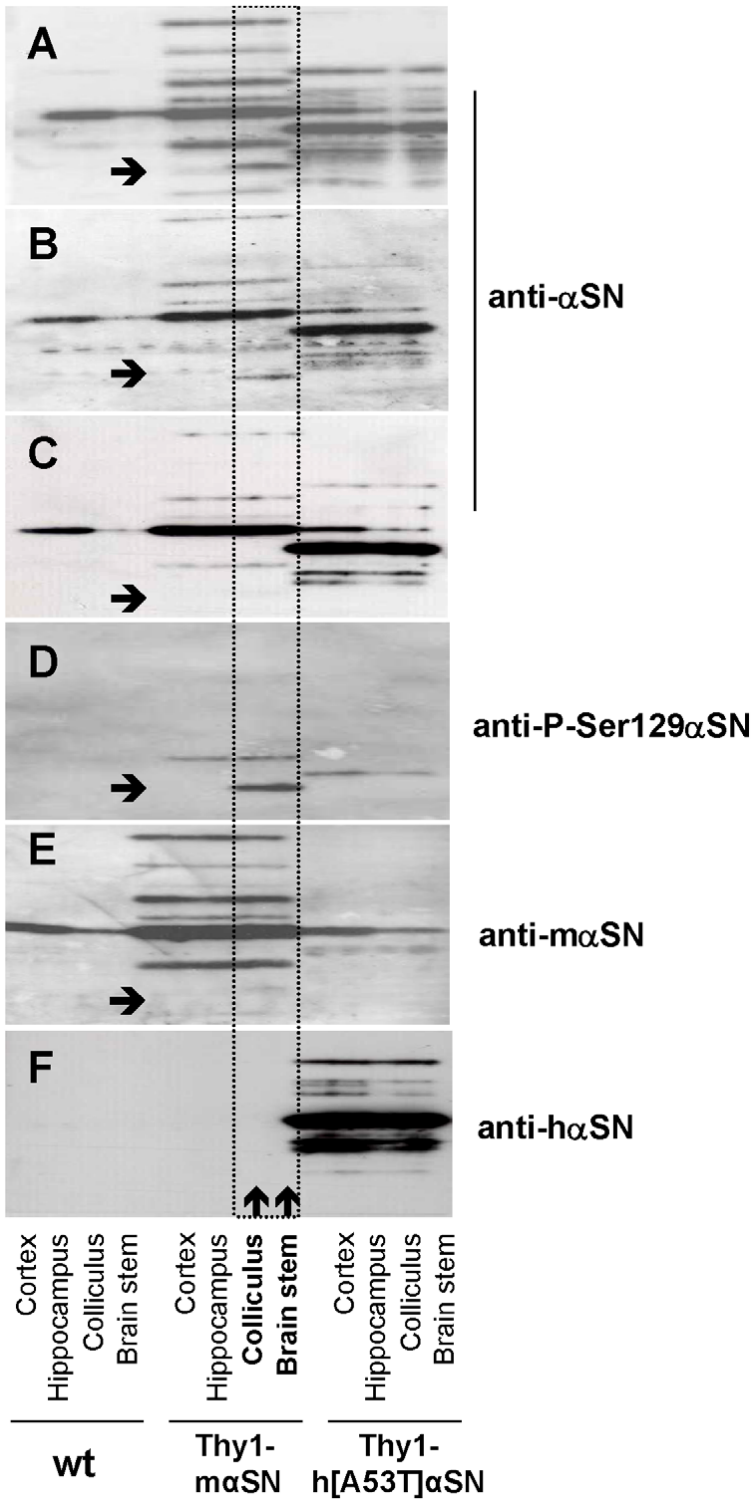
Isoelectric focusing Western blot analysis of αSN isoforms. (**A–F**) Each panel shows the immunoblot analysis of 12 brain protein samples fractionated by isolectric focusing on a pH 4.5–5.4 gradient. Bars cover four lanes representing the same mouse. Lanes 1–4, wildtype (wt) mouse brain. Lanes 5–8, Thy1-mαSN mouse brain. Lanes 9–12, Thy1-h[A53T]αSN mouse brain [6]. Lanes 1, 5, and 9 are protein homogenates from cortex. Lanes 2,6,10, hippocampus. Lanes 3,7,11, colliculus. Lanes 4,8,12, brainstem. After isolelectric focusing, αSN isoforms were visualized using the following 4 different antibodies: (**A**) anti-αSN (Syn-1, S63320, Transduction Labs), (**B**) anti-αSN (4D6, Abcam), (**C**) anti-αSN (5038, Chemicon), (**D**) anti-P-Ser129 αSN (WAKO), (**E**) anti-mouse αSN-specific Ab [5] and (**F**) for comparison, an anti-human αSN-specific antibody that fails to recognize mouse αSN (Syn211, Zymed). The arrows indicate a novel αSN isoform specific to colliculus and brainstem.

Isoelectric focusing Western blotting using several antibodies were performed to characterize the αSN isoforms expressed in the brain of Thy1-mαSN mice (**Figure 7**). We found a novel αSN isoform specific to colliculus and brainstem, the two regions with extensive ubiquitin pathology (**Figure 7A,B,D**). Importantly the novel P-Ser129αSN isoform is not detected using αSN antibodies targeting the C-terminus (**Fig. 7C,E**) and additionally not present in previously characterized mouse lines expressing hαSN [6] (**Figure 7F**).

Summarizing the expression of mαSN isoforms in mice showed pronounced ubiquitin immunopathology in spinal cord including a novel αSN isoform. Additionally, we observed a strong αSN pathology in the spinal cord accompanied with axonal degeneration. These findings were followed by signs of presynaptic degeneration with reduced neurofilament staining in neuromuscular junction synapses. Interestingly, hippocampal neurons showed strong αSN accumulation but no ubiquitination in contrast to spinal cord motor neurons. Furthermore, we showed that few neurons in the cortex display an intriguing staining pattern of ubiquitin and phosphorylated mαSN, suggesting that these posttranslational modifications play a role in trafficking and localization of αSN.

## Discussion

Transgenic animals are considered excellent preclinical models to study α-synuclein (αSN) disease pathophysiology and test therapeutic strategies. Here we show that wildtype murine αSN can induce pathological changes in mouse brain closely resembling those observed in post-mortem human PD and DLB brains. These transgenic mice are very similar to those over-expressing human wildtype or the familial PD point-mutated A53T αSN [6]. Van der Putten et al. used the same Thy1 promoter to drive comparable αSN levels in similar brain regions compared to our Thy1-mαSN transgenic mice. This is very intriguing since for the first time we show that murine αSN as well as human αSN can be pathogenic in neurons *in vivo*.

Interestingly, profound neuropathological changes could be detected solely in spinal cord, brainstem and cerebellum (after six months of age). Forebrain areas were always histopathologically unaffected despite a strong murine αSN over-expression. We show that neurons in the forebrain (like CA1 pyramidal cell) displayed the same strong somatic αSN staining as cells in the brainstem and cerebellum. Thus, this abnormal somatic accumulation of αSN does not account for the difference of histopathology observed for the different brain areas leading to the conclusion that rather the endogenous ‘normal’ αSN expression pattern might be responsible for the different neuronal vulnerability. For example, in wildtype (wt) animals, αSN is highly expressed in forebrain regions compared to brainstem and cerebellum. This suggests that neurons with low endogenous αSN levels are more sensitive to over-expression of αSN at a certain age. Unfortunately, the short life expectancy of Thy1-mαSN mice hindered a possible histopathology in the forebrain later in development. A forebrain-specific expression (e.g. αCamKII) could unravel this issue.

In solution αSN is natively unfolded, whereas the acidic phospholipids are α-helical in nature. In cells, the structure of αSN is highly dynamic forming equilibrium between oligomers, β-sheets and large aggrosomes that is context dependent [15], [16]. We observed in brains of Thy1-mαSN (<6 months of age) several isoforms of mαSN whereas one of it was specifically restricted to brainstem and colliculus, areas of late-onset neuropathology. This specific isoform was Ser129-phosphorylated (P-Ser129αSN) and notably, could be detected before the appearance of any immunopathology. Moreover, this specific isoform could not be detected with antibodies targeting the C-terminus, which might be due to the heavy phosphorylation of this isoform that hindered antibody binding. Brain areas without any late-stage histopathology were devoid of this isoform, while the abundance of other isoforms was unchanged. The identification of the sequence of this isoform might give more insights about its function and its involvement in the neuropathological process.

However, we could not observe any brain area specific isoform in mice over-expressing hαSN which could merely be related to the antibodies used. Thus, the P-Ser129αSN isoform could be attributed to the brain area-specific late-onset neuropathology observed in Thy1-mαSN mice. Soluble forms of αSN, αSN fibrils and protofibrils, soluble protein complexes of αSN with 14-3-3 protein, phosphorylated, nitrosylated, and ubiquitinated αSN species have all been implicated in neurotoxicity [2], [17].

There is a cause-and-effect relationship between Ser129-phosphorylation of αSN and the disease. Noted in several animal models was an accumulation of Ser129-hyperphosphorylated, nitrosylated, ubiquitinated, and/or fragmented αSN species [6], [7], [8], [9], [13], [18]. P-Ser129αSN yields more insoluble sediments and oligomers as compared to its non-phosphorylated counterpart. Moreover, the αSN pathology in human brain, transgenic mouse brain and transgenic fly neurons are enriched in Ser129-hyperphosphorylated αSN [10], [13], [19], [20]. Our current findings suggest that Ser129-phosphorylation of αSN by itself is not sufficient to cause αSN pathology in neurons *in vivo*. We found that over-expression of wildtype mαSN greatly enhanced levels of P-Ser129αSN in different brain regions, but only some of these regions show cellular hallmarks of αSN pathology. Posttranslational modification of Ser129 by phosphorylation seems therefore part, but not the whole reason to convert the αSN molecule into toxic entities. Transgene expression of a phosphorylation-defective Ser129 substitution mutant Ala129 is now needed to experimentally confirm this hypothesis.

In several transgenic models αSN causes an impairment of neuronal function and the development of αSN micro and/or macro aggregates (amorphous, granular and/or fibrillar). Some have been shown to be proteinase-resistant αSN aggregates [13]. The pathology has been observed in synapses, axons, dendrites and neuronal cell somata. Occasionally, there was also inclusion formation in glia. However, in the majority of transgenic rodent models αSN aggregates appeared non-fibrillar, granular and/or amorphous at the electron microscopic level [13]. This is in line with our observations of non-fibrillary amorphous aggregates. Biochemical extractions did show an age-dependent shift into buffer-insoluble fractions, but no detergent resistance of αSN in Thy1-maSN mouse brains. Rarely, investigators have reported fibrillar αSN structures similar to filamentous αSN observed in human samples [7], [13]. Altogether the findings seem to suggest that fibrillar, as well as other types of αSN aggregates, are associated with pathophysiological effects of αSN *in vivo* [6], [8], [9], [18].

Phosphorylated mono- and di-ubiquitinated αSN forms exist in human brains with α-synucleinopathy suggesting that phosphorylated αSN is targeted to mono- and di-ubiquitination [4]. UCH-L1 and/or Parkin mediated ubiquitination of αSN could control its function, catabolism/stability, localization and interaction with other molecules and levels of toxic protofibrils, although knock-out of UCH-L1 in Thy1-mαSN had no effect on αSN metabolism and localization (unpublished observation). Ubiquitination in Thy1-maSN transgenic mice was evident along with the other histopathologies in every mouse that we analysed and was restricted to brainstem, cerebellum and spinal cord. This is in contrast to mice over-expressing the human form of αSN showing ubiquitination only sporadically [6]. The amino-acid sequence of human and mouse αSN are very similar and differ only at seven positions. This includes position 53, where in wildtype mouse αSN the amino-acid threonine instead of alanine is present. The human pathogenic mutation A53T hence naturally exists in mouse and thus, cannot account for the difference observed in ubiquitination in mice over-expressing wildtype mouse and human A53T point-mutated αSN. This suggests that rather the six other amino-acid differences between human and mouse that are located downstream of position 53 guide the ubiquitin pathology. In human brains, ubiquitinated structures represent mainly classical Lewy bodies and Lewy neurites [21]. However, the brain areas positive for strong ubiquitination in our mice did not display any enhanced degree of αSN aggregation. This indicates that ubiquitination might not be required for the formation αSN inclusions as described elsewhere [22].

Mice over-expressing murine wildtype αSN showed no early motor deficits aside from minor motor learning impairment. Motor impairments are not obvious until 6 months of age that coincide with a rapid decline in health, resulting in death of the animal. This is in sharp contrast to mice over-expressing human αSN displaying early-onset motor deficits. Hence, murine αSN in 6-fold higher dose unlike human αSN somehow might not interfere with normal neuronal function in early development.

All Thy1-mαSN animals analysed developed alongside these motor deficits degeneration of the NMJ, astrogliosis, microgliosis, axonal and ubiquitin pathology. Classical Lewy body-like structures were not observed. Unfortunately, the Thy1 promoter fails to express in dopaminergic neurons (data not shown) and thus, no pathology in the substantia nigra pars compacta could be observed. Although, these cells in the human brain are most sensitive to develop Lewy pathology, and the resulting nigral lesions are primarily involved in the clinical symptoms of PD [23], [24], [25], [26], extranigral Lewy pathology is very common in PD and LBD brains [23], [27], [28], [29].

Moving towards disease-modifying therapies requires a general understanding of the role of (epi)genetic and environmental factors. Moreover, insights into the presymptomatic/symptomatic changes and of the molecular identity of the culprit(s) and pathway(s) that drive disease process are necessary. We also need diagnostic tools, biomarkers and translational animal models that mimic αSN-induced pathophysiological changes and allow testing of the effects of drugs, antibodies, genes and RNAi that halt and/or reverse disease. Human mutant, human wt, and mouse wt αSN drive disease pathophysiology and loss of neuronal cell function. These transgenic mice display many hallmark features of human pathology and provide means to address fundamental aspects of disease pathophysiology, explore surrogate markers, test therapeutic strategies with behavioural and biochemical read-outs and provide a good model for extra-nigral α-synucleinopathy.

## Materials and Methods

### Statement on Animal Health

All experiments were carried out in accordance with authorization guidelines of the Swiss Federal and Cantonal veterinary offices for care and use of laboratory animals. Studies described in this report were approved by the Swiss Cantonal veterinary office and performed according to Novartis animal license number 2063.

### Transgenic mice

Wildtype mouse α-synuclein cDNA (531 bp) was PCR amplified (2 min. 93°C; 3 cycles of 15 sec, 93°C; 30 sec, 55°C; 30 sec, 72°C; 2 cycles of 15 sec, 93°C; 30 sec, 60°C; 30 sec, 72°C; and 30 cycles: 15 sec, 93°C; 30 sec, 66°C; 30 sec, 72°C; oligonucleotides GGGAGCCGTGTGGAGCAAAAATAC and TGGGCACATTGGAACTGAGCACTT) from 20 ng C57BL/6 brain cDNA (in-house C57BL/6 strain) and cloned into pMOSBlue (Amersham, UK). The identity of the cDNA was confirmed by sequencing and the corresponding blunted (Klenow) cDNAs (NotI) inserted into the blunted (XhoI site) Thy1 cassette [6], [30]. For microinjection, linear NotI DNA fragments comprising transgene without plasmid vector sequences were isolated. Injection was into homozygous C57BL/6 mouse eggs. Genotyping was performed by PCR (oligonucleotides HP45 Thy1 sense (5′-ACA CCC CTA AAG CAT ACA GTC AGA CC-3′) and HP42 aSN antisense (5′- TGG GCA CAT TGG AAC TGA GCA CTT-3′); 5 min 95°C, 35 cycles of 30 s 95°C, 40 sec 62°C, 40 sec 72°C, 10 min. at 72°C) with standard procedures using column-purified (Qiagen) tail DNA. Expected band size of the transgene: 1200 bp.

### Northern blot analysis

Northern blot analysis was carried out with total brain RNA (TriZol method; 10 µg loaded per gel lane). Blots were probed with a full length (449 bp) mouse α-synuclein cDNA using standard procedures.

### *In Situ* hybridization

The spatial distribution pattern of transgene versus endogenous α-synuclein expression was determined by in situ hybridization as described before [6] using cRNA derived from a 449 bp complete coding cDNA template of mouse α-synuclein.

### Western blot and isoelectric focusing (IEF) analysis

For standard SDS-PAGE Western blot analysis, 14.000× g supernatant fractions were used of half-brain homogenates (homogenized in 2 ml E-buffer; 50 mM Tris-HCl, pH 7.4, 1% NP-40, 0.25% Na-deoxycholate, 150 mM NaCl, 1 mM EDTA, a cocktail of protease inhibitors (Boehringer Mannheim), and left on ice for 30 min). 50 µg protein was loaded per lane and separated on 15% SDS-PAGE. After blotting and blocking non-specific binding, membranes were incubated with monoclonal anti-α-synuclein antibody (1∶1000; Syn-1, S63320, Transduction Laboratories), followed by HRP-conjugated anti-mouse IgG (1∶1000, RPN2108, Amersham), and ECL western blotting detection reagents (RPN2108, Amersham).

For solubility assays, pooled frozen brainstems (matching 0.2 g starting wet weight) were homogenized in 10 volumes buffer A (50 mM Tris-HCl, pH 7.5, 1 mM EDTA, 1 mM dithiothreitol) using a Teflon potter. The homogenate was cleared by centrifugation for 10 min at 1000× *g*. Cleared homogenates were centrifuged for 20 min at 350,000× *g*. The buffer-insoluble pellets were resuspended in buffer A+1% Triton X-100, followed by another centrifugation for 20 min at 350,000× *g*. All steps were carried out on ice. Preparation of purifed recombinant αSN control protein was described elsewhere [31]. Samples were boiled in Lämmli buffer and subjected to denaturing 12.5% polyacrylamide gel electrophoresis. Western blots were prepared and probed with monoclonal anti-αSN (1∶250; Transduction Laboratories). Ponceau red staining of the polyvinylidene fluoride membranes confirmed equal loading.

For IEF Western blot analysis, brain samples were homogenized in a buffer [32] containing 4% CHAPS, 7 M urea, 2 M thiourea, 10 mg/mL dithiothreitol, and 1% carrier ampholytes (pH 3–10; Pharmalytes; Amersham Pharmacia Biotech, Uppsala, Sweden). Isoelectric focusing on immobilized pH gradient plates (pH range 4.5–5.4, Amersham Pharmacia), transfer to PVDF membranes, and immunodetection was carried out as described [33]. The protocol included a trichloroacetic acid wash step and formaldehyde fixation of the transferred proteins [33]. Protein extracts were adjusted to 10 µg/µl and 1 µl loaded per lane. Bound antibodies were detected by peroxidase conjugates against rabbit IgG (1∶2000, NA934V, GE Healthcare), mouse IgG (1∶2000, NA931V, GE Healthcare), or an alkaline phosphatase conjugate against sheep IgG (1∶5000; Jackson ImmunoResearch 313 055 033). ECL Western blotting detection reagents for peroxidase (Amersham Pharmacia RPN2109) or Lumi-Phos WB for phosphatase (Pierce 34150) were used for visualization. Antibodies used: mouse anti-αSN (Syn-1, S63320, Transduction Labs), mouse anti-αSN (4D6, Abcam), rabbit anti-αSN (5038, Chemicon), mouse anti-P-Ser129αSN (WAKO), anti-mouse αSN-specific Ab [5] and anti-human αSN-specific antibody (Syn211, Zymed).

### Immunohistochemistry

Mice (age 1.5–8 months) were perfused transcardially with 0.01 M phosphate buffered saline (PBS) followed by 4% paraformaldehyde in PBS. Brain, spinal cord and hind limb muscle were embedded in paraffin and cut as 4 µm thick sections or 25 µm vibratome sections were cut of the brain for free floating immunohistochemical staining using mouse anti-α-synuclein antibodies (Syn-1, 1∶500; S63320, Transduction Laboratories and 4D6, 1∶800; Abcam), rabbit anti-α-synuclein (1∶1000, AB5038, Chemicon), mouse anti-P-Ser129 α-synuclein (1∶20000, WAKO), biotinylated anti-mouse IgG (1∶500; E0464, Dako) and avidin-biotin peroxidase method (Elite standard kit SK6100, Vector). Deparaffinized sections were used for Campbell Switzer silver staining and immunostaining with rabbit anti-ubiquitin Ig fraction (1∶200; Z0458, Dako). Antigenity was enhanced by treating paraffin sections with concentrated formic acid for 5 min and microwave heating at 90°C for 60 min before incubation with anti-α-synuclein; microwave heating at 90°C for 30 min before anti-GFAP and anti-phosphotyrosine anti-ubiquitin treatment at 37°C, 30 min. Non-specific binding sites were blocked using normal serum. Bound antibody was visualized using avidin-biotin peroxidase method (Elite standard kit SK6100, Vector) and DAB substrate (1718096 Boehringer), Vector VIP substrate (SK-4600, Vector) or with fluroescence-labeled secondary antibodies (FITC or Cy3-labeled goat anti-mouse, FITC or Cy3-labeled goat anti-rabbit, Jackson ImmunoResearch).

Immunostaining and confocal analysis of neurofilament and synaptophysin at neuromuscular junctions (NMJs) were as follows. Mice were killed by anaesthesia. Extensor digitorum longus (EDL) and soleus muscle were stained with Alexa Fluor 488-labeled α-bungarotoxin (1∶200, Molecular Probes) for 30 min, washed with PBS (3×15 min) and fixed with 1.5% paraformaldehyde for 10 min. Muscles were teased into approximately 20 thin bundles and permeabilized with 1% Triton X-100 in PBS for 1 h. Bundles were treated with 100 mM glycine in PBS, followed by “blocking solution” of 1% BSA in PBS, for 30 min. Then, they were incubated overnight at 4°C with a mixture of primary antibodies against synaptophysin (1∶200, DAKO) and neurofilament (1∶1000, MAB 1621, Chemicon) in blocking solution and washed three times 1 h in blocking solution. The bundles were incubated with a mixture of Cy3-labeled goat anti-mouse IgG (1∶1000, Jackson ImmunoResearch) and Cy5-labeled goat anti-rabbit IgG (1∶500, Jackson ImmunoResearch) in blocking solution for 45 min at rommtemperature. After washing three times for 1 h with blocking solution, bundles were mounted on glass slides using Citifluor (Plano), and examined with a confocal microscope (Leica TCN NT).

### Immunoelectron and electron microscopy

For immunoelectron microscopy, transgenic and wildtype C57Bl/6 mice were perfused transcardially with a mixture of 1.5% picric acid, 0.1% glutaraldehyde, and 4% paraformaldehyde in 0.1 Mphosphate buffer, pH 7.4. Vibratome sections were stained free-floating with antibody to P-Ser129 α-synuclein (1∶2000, anti-P-Ser129 α-synuclein, WAKO) dehydrated in ascending series of ethanol and acetone, and flat-embedded between glass slide and coverslip in Embed-812 (Electron Microscopy Sciences). Fragments of the spinal cord were then dissected out and ultra-thin sections were cut from the tissue surface, and these were mounted on copper grids and analyzed with a microscope (EM900, Zeiss). For conventional electron microscopy, mice were anesthetized and perfused transcardially with cold saline, followed by 4% paraformaldehyde and 0.1% glutaraldehyde in 0.1 M cacodylate buffer. Small tissue blocks were cut out from brainstem and spinal cord, immersion-fixed for 12 h at 4°C in the same buffer, and epoxy-embedded, and ultra-thin sections were prepared and placed on 200-mesh copper grids for staining with uranyl acetate and lead citrate.

### Behavior

#### Rotarod

To measure motor coordination mice were placed on a computerized treadmill (TSE rotarod system, Germany). The rotarod program consists of accelerating running speed from 5 rpm to 36 rpm in 5 min. Rotarod performance was assessed by evaluating the two best trials out of three performed in one day. The 3-step rotarod consists of a modified rotarod program of three different running speeds (12 rpm, 24 rpm and 36 rpm) each for 30 sec with intervals of acceleration lasting for 10 sec. Starting speed is 4 rpm. Rotarod performance was assessed by evaluating the two best trials out of three performed in one day.

#### Grip strength

To measure forelimb grip strength, mice are allowed to grasp a handle connected to a force-measuring device (San Diego Instruments, USA) and then pulled back with their tails until they release the handle. The best out of four consecutive trials is evaluated.

#### Open field

To measure exploratory behavior (pattern and activity), mice were placed in an open field box (70 cm×70 cm, height of walls: 30 cm) subdivided into nine quadrants with one middle quandrant. The horizontal distance travelled during 5 min was recorded by an EthoVision 3.0 system (Noldus, The Netherlands). In addition the number of rearings was determined by visual inspection.

#### Actimeter

Recordings were made in automated circular corridors (Imetronic, France) for 48 h. These corridors with a radius of 4.5 cm and a width of 5.3 cm were equipped with 4 photocells, equidistant of 7 cm and 45° from each other, connected to an electronic interface, itself connected to a computer. Motor activity corresponds to the number of photocell interruption per time unit (20 min) and locomotor activity corresponds to the number of quarter turns corresponding to the successive interruption of two photocells. The dark and light phases lasted 12 h each.

#### Dark/light box

The dark/light box consists of a dark and a bright compartment. Mice were placed in the bright compartment and given the opportunity to move to the dark box for 5 min. Parameters measured by EthoVision 3.0 (Noldus, The Netherlands) were the time spent in the bright compartment and the latency of first entry to the dark compartment. The number of transitions and the latency of first exit back to the bright compartment were measured visually.

#### Elevated plus-maze

The elevated plus-maze (80 cm from the floor) consists of four arms (length: 27 cm) arranged in right angles to each other. Two opposite arms have walls (height: 15 cm) and the two others are open. Mice are placed in the middle and are allowed to move freely for 5 min. The time spent in the open arms is recorded by EthoVision 3.0 (Noldus, The Netherlands) and the number of entries to open arms by visual inspection.

### Maintenance

The animals were housed in a temperature-controlled room that was maintained on a 12 h light/dark cycle. Food and water were available *ad libitum*.

## Supporting Information

Figure S1 **Bodyweight and behaviour analysis of Thy1-h[A53T]αSN transgenic mice.** (**A**) Body weight assessment in Thy1-h[A53T]αSN (+/T, black) and littermate wildtype (wt) controls (white); n = 12. Thy1-h[A53T]αSN mice show reduced behavior activity as measured by total distance (**B**) and velocity (**C**) in the open field paradigm. (**D**) Strong reduction of forelimb grip strength in Thy1-h[A53T]αSN compared to wt mice. (**E**) Measurement of the performance on the 3-step rotarod reveals strong locomotor impairment of Thy1-h[A53T]αSN mice. (**F**) Quantification of the latency of the first entry to the dark and of the time spent in the lit compartment in the dark/light box in 5 min. (**G**) Measurement of the latency of the first entry to the dark and of the time spent in the lit compartment in the dark/light box in 5 min. Age of the animals: 2–3 months. Data are shown as mean ± SEM (n = 10). * p<0.05; ** p<0.01; *** p<0.001.
(TIF)

Figure S2 **Increased αSN accumulation in Thy1-mαSN transgenic mice.** (**A–D**) Prominent αSN immunoreactivity in CA1 (**A,C**) and cerebellar nuclei (**B,D**) at different ages (1.5 and 3 months old) compared to low αSN immunoreactivity in wildtype (wt) littermates (3 months) (**E,F**).
(TIF)

Figure S3 **Transgene and endogenous mαSN protein expression in hippocampus.** (**A–D**) Immunofluorescence images of mαSN protein detected in 25 µm thick free-floating sagittal hippocampal sections of a wildtype (wt) mouse (**A**), a Thy1-mαSN mouse (**B**), a αSN KO mouse (**C**) and a Thy1-mαSN transgene after crossing into the αSN knock-out (KO) genetic background (**D**).
(TIF)

Figure S4 **Thy1-mαSN transgenic mice show increased inflammation.** (**A,B**) Immunoperoxidase stained sagittal sections of GFAP from wildtype (wt) (**A**) and Thy1-mαSN (+/T) (**B**) mice. (**C–K**) high power magnification of pontine nuclei stained GFAP (**C,D**), Iba1 (**F,G**) and Campbell (**I,J**) and the quantification respectively (**E,H,K**). Data are shown as mean ± SEM (n = 6); ** p<0.01; *** p<0.001.
(TIF)
