# Supplementary figures and images for: Neuropathology in Mice Expressing Mouse Alpha-Synuclein
Source: PLoS One. 2011 Sep 26;6(9):e24834. doi: 10.1371/journal.pone.0024834 (PMC3180287; doi:10.1371/journal.pone.0024834)

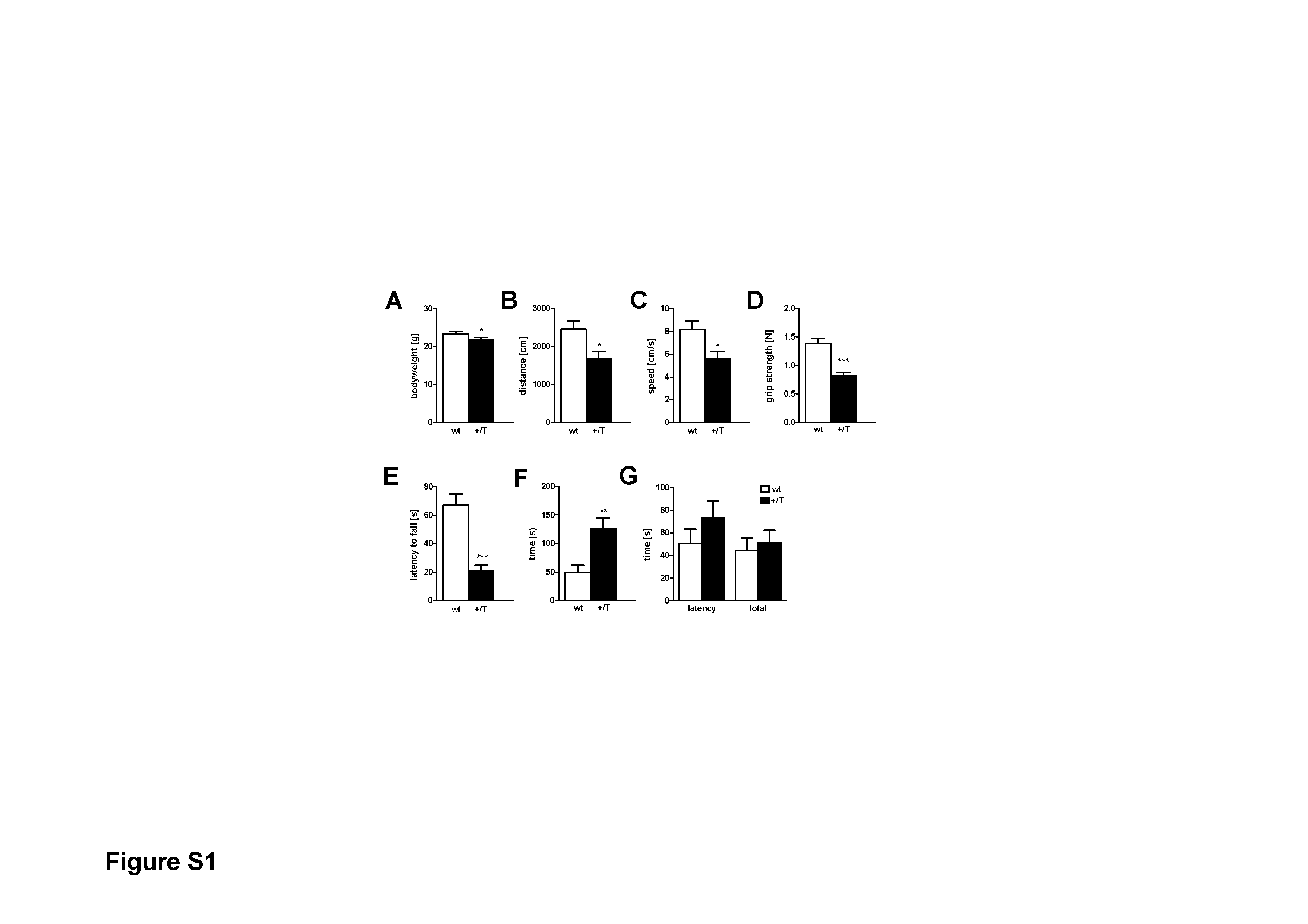

Supplement: Figure S1 — Bodyweight and behaviour analysis of Thy1-h[A53T]αSN transgenic mice. (A) Body weight assessment in Thy1-h[A53T]αSN (+/T, black) and littermate wildtype (wt) controls (white); n = 12. Thy1-h[A53T]αSN mice show reduced behavior activity as measured by total distance (B) and velocity (C) in the open field paradigm. (D) Strong reduction of forelimb grip strength in Thy1-h[A53T]αSN compared to wt mice. (E) Measurement of the performance on the 3-step rotarod reveals strong locomotor impairment of Thy1-h[A53T]αSN mice. (F) Quantification of the latency of the first entry to the dark and of the time spent in the lit compartment in the dark/light box in 5 min. (G) Measurement of the latency of the first entry to the dark and of the time spent in the lit compartment in the dark/light box in 5 min. Age of the animals: 2–3 months. Data are shown as mean ± SEM (n = 10). * p<0.05; ** p<0.01; *** p<0.001. (TIF) [file pone.0024834.s001.tif]

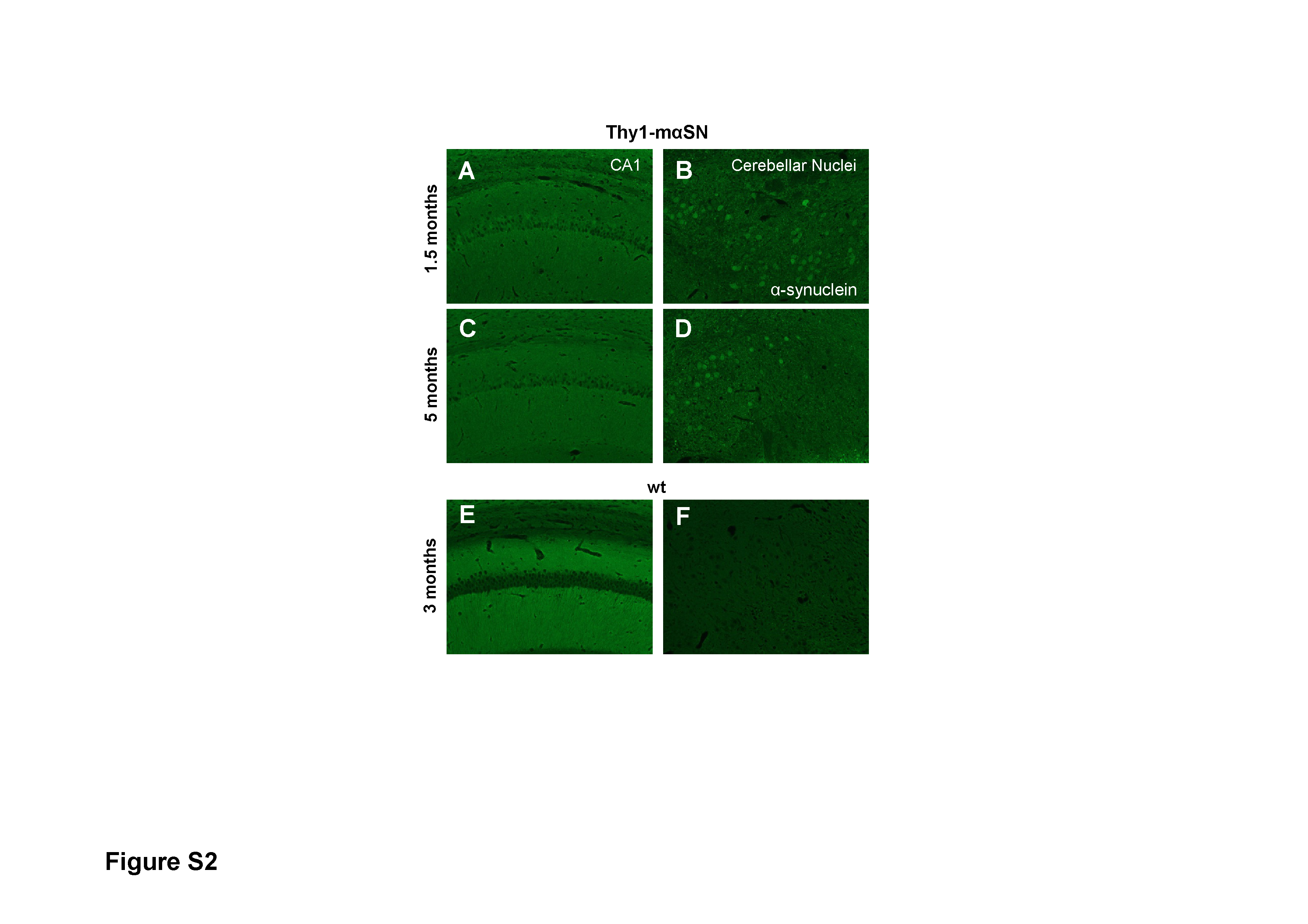

Supplement: Figure S2 — Increased αSN accumulation in Thy1-mαSN transgenic mice. (A–D) Prominent αSN immunoreactivity in CA1 (A,C) and cerebellar nuclei (B,D) at different ages (1.5 and 3 months old) compared to low αSN immunoreactivity in wildtype (wt) littermates (3 months) (E,F). (TIF) [file pone.0024834.s002.tif]

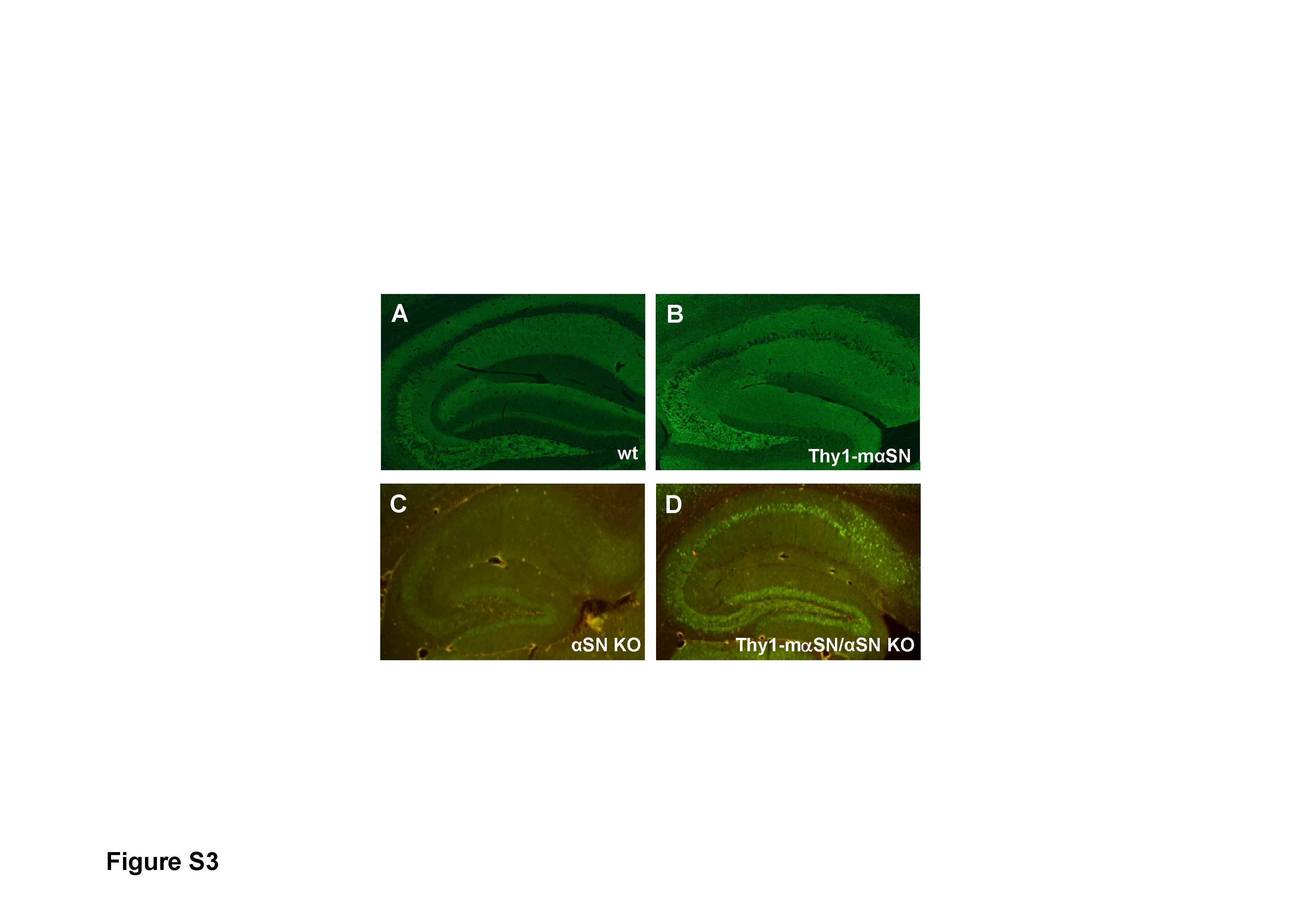

Supplement: Figure S3 — Transgene and endogenous mαSN protein expression in hippocampus. (A–D) Immunofluorescence images of mαSN protein detected in 25 µm thick free-floating sagittal hippocampal sections of a wildtype (wt) mouse (A), a Thy1-mαSN mouse (B), a αSN KO mouse (C) and a Thy1-mαSN transgene after crossing into the αSN knock-out (KO) genetic background (D). (TIF) [file pone.0024834.s003.tif]

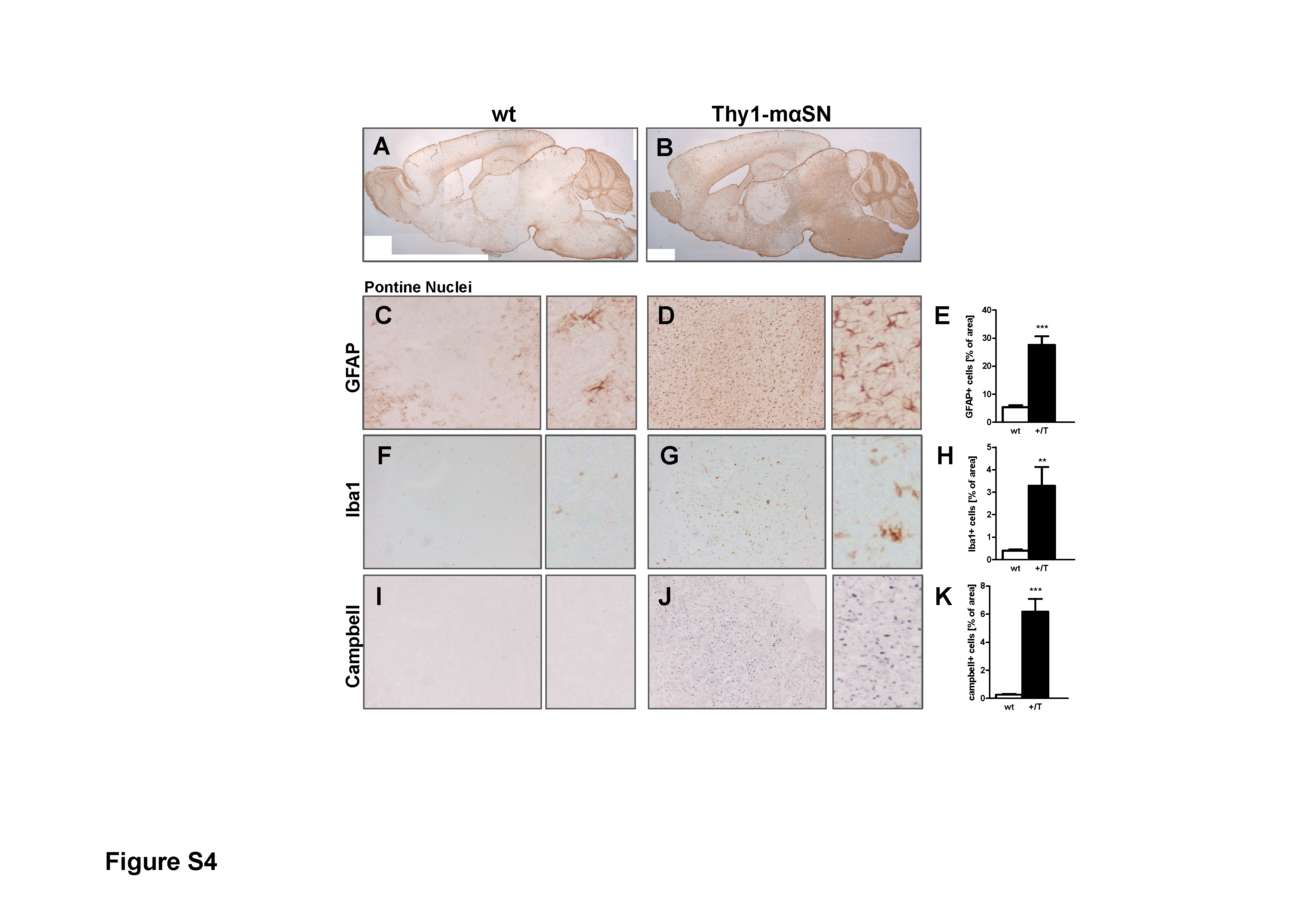

Supplement: Figure S4 — Thy1-mαSN transgenic mice show increased inflammation. (A,B) Immunoperoxidase stained sagittal sections of GFAP from wildtype (wt) (A) and Thy1-mαSN (+/T) (B) mice. (C–K) high power magnification of pontine nuclei stained GFAP (C,D), Iba1 (F,G) and Campbell (I,J) and the quantification respectively (E,H,K). Data are shown as mean ± SEM (n = 6); ** p<0.01; *** p<0.001. (TIF) [file pone.0024834.s004.tif]
